# Next-generation sequencing reveals broad down-regulation of microRNAs in secondary progressive multiple sclerosis CD4+ T cells

**DOI:** 10.1186/s13148-016-0253-y

**Published:** 2016-08-27

**Authors:** Katherine A. Sanders, Miles C. Benton, Rod A. Lea, Vicki E. Maltby, Susan Agland, Nathan Griffin, Rodney J. Scott, Lotti Tajouri, Jeannette Lechner-Scott

**Affiliations:** 1 Faculty of Health Sciences and Medicine, Bond University, Robina, Queensland 4226 Australia; 2 Centre for Information-Based Medicine, Hunter Medical Research Institute, Newcastle, New South Wales 2305 Australia; 3 School of Biomedical Sciences and Pharmacy, University of Newcastle, Newcastle, New South Wales 2308 Australia; 4 Institute of Health and Biomedical Innovation, Genomics Research Centre, Brisbane, Queensland 4059 Australia; 5 Department of Neurology, Division of Medicine, John Hunter Hospital, Locked Bag 1, Hunter Region Mail Centre, Newcastle, NSW 2310 Australia; 6 Division of Molecular Genetics, Pathology North, Newcastle, New South Wales 2305 Australia; 7 School of Medicine and Public Health, University of Newcastle, Newcastle, New South Wales 2308 Australia

**Keywords:** Multiple sclerosis, Secondary progressive, MicroRNAs, Immunology, CD4+ T cells, Next-generation sequencing

## Abstract

**Background:**

Immunoactivation is less evident in secondary progressive MS (SPMS) compared to relapsing-remitting disease. MicroRNA (miRNA) expression is integral to the regulation of gene expression; determining their impact on immune-related cell functions, especially CD4+ T cells, during disease progression will advance our understanding of MS pathophysiology. This study aimed to compare miRNA profiles of CD4+ T cells from SPMS patients to healthy controls (HC) using whole miRNA transcriptome next-generation sequencing (NGS). Total RNA was extracted from CD4+ T cells and miRNA expression patterns analyzed using Illumina-based small-RNA NGS in 12 SPMS and 12 HC samples. Results were validated in a further cohort of 12 SPMS and 10 HC by reverse transcription quantitative polymerase chain reaction (RT-qPCR).

**Results:**

The ten most dysregulated miRNAs identified by NGS were selected for qPCR confirmation; five (miR-21-5p, miR-26b-5p, miR-29b-3p, miR-142-3p, and miR-155-5p) were confirmed to be down-regulated in SPMS (*p* < 0.05). *SOCS6* is targeted by eight of these ten miRNAs. Consistent with this, *SOCS6* expression is up-regulated in SPMS CD4+ T cells (*p* < 0.05). This is of particular interest as *SOCS6* has previously been shown to act as a negative regulator of T cell activation.

**Conclusions:**

Ninety-seven percent of miRNA candidates identified by NGS were down-regulated in SPMS. The down-regulation of miRNAs and increased expression of *SOCS6* in SPMS CD4+ T cells may contribute to reduced immune system activity in progressive MS.

**Electronic supplementary material:**

The online version of this article (doi:10.1186/s13148-016-0253-y) contains supplementary material, which is available to authorized users.

## Background

Multiple sclerosis (MS) is an autoimmune disease characterized by multifocal inflammatory attacks in the CNS [1]. In the relapsing-remitting (RRMS) stage of the disease, CD4+ T cells are among the primary infiltrators moving from the periphery, through the blood-brain barrier, and into the CNS [2]. These cells then initiate an immune response that results in localized demyelination and corresponding symptoms. The later stage of MS, secondary progressive (SPMS), is characterized by compounding neurodegeneration and increasing disability; however, the relevance of inflammation is unclear [3]. As key regulators of gene expression, microRNAs (miRNAs) may be affecting the immune-related functions of CD4+ T cells in SPMS and may help to elucidate the actions of these cells in SPMS.

MiRNAs are short, non-coding RNA molecules (~22 bp) that regulate gene expression at the posttranscriptional stage by targeting the 3′ untranslated region of target genes. Their small size and stable structure make them ideal biomarkers. In recent years, miRNA expression patterns in MS have been the focus of numerous studies, many of which have concentrated on using miRNAs as biomarkers for diagnosis and prognosis [4]. These studies predominantly use easily acquired (and often highly heterogeneous) samples such as whole blood, peripheral blood mononuclear cells (PBMCs), serum, and plasma. Numerous dysregulated miRNAs have been identified, however which cell types are actually responsible for differing miRNA profiles, and the consequences of altered miRNA expression is not clear in many studies. Furthermore, it is likely that these heterogeneous samples are masking the signal of differentially expressed miRNA in specific cell subtypes. To overcome this, we have focused on CD4+ T cells in this study on SPMS.

Next-generation sequencing (NGS) allows for stringent examination of cell-specific miRNA expression profiles as well as discovery of previously uncharacterized miRNAs. Here, we have used small-RNA NGS analysis of CD4+ T cells from SPMS patients and healthy controls (HC). The total coverage approach of NGS generates expression information on all small RNA species including all known and novel miRNAs, as well as other small RNA species (isomiRs and snoRNAs)—a clear advantage over microarray and candidate approach assays. Three previous studies in MS have used NGS to effectively identify miRNA expression profiles in the whole blood [5, 6], serum [6], and PBMCs [7] from RRMS patients. However, NGS techniques have not been used for specific cell types or in SPMS samples.

The miRNA expression profile of CD4+ T cells, either as instigating molecules or by-products of erroneous molecular mechanisms, will provide insight into the function of these cells in SPMS. Here, we used NGS to provide a comprehensive analysis of the miRNA expression profiles of CD4+ T cells from SPMS patients and healthy controls (HC) and confirmed these results using targeted expression assays.

## Methods

### Sample collection

Whole blood was collected at a single study center from an initial cohort of 12 SPMS patients and 12 HC and a replication cohort of 12 SPMS and 10 HC. All patients were diagnosed with SPMS according to the McDonald criteria [8] and demonstrated EDSS progression without evidence of relapse in the 24 months prior to collection. Controls were age (±5 years) and gender matched (Table 1). The SPMS patient group was free of MS-specific treatments for a minimum period of 6 months prior to collection. Samples were collected at the John Hunter Hospital, and laboratory work was conducted at the Hunter Medical Research Institute, Newcastle.

**Table 1:** Details of SPMS and healthy control individuals

	Next generation sequencing		Replication cohort	
	SPMS	HC	SPMS	HC
Number	12	12	12	10
Female	9	9	8	5
Age in years (mean ± SD)	60.2 ± 8.3	61.3 ± 9.5	61.4.0 ± 6.5	60.1 ± 5.9
EDSS (mean ± SD)	6.9 ± 0.9	NA	5.9 ± 1.0	NA
Active SPMS	3	NA	4	NA
Disease duration in years (mean ± SD)	25.6 ± 11.1	NA	18.3 ± 6.5	NA
Progression duration (mean ± SD)	10.8 ± 8.1	NA	8.9 ± 6.2	NA

*EDSS* expanded disability status scale, *SD* standard deviation, *NA* not applicable

### Blood sample processing

PBMCs were isolated from 45 mL of heparinized whole blood by density gradient centrifugation on lymphoprep (Axis-Shield PoC AS, Norway). CD4+ T cells were enriched from the PBMCs using EasySep magnetic negative selection according to the manufacturer’s protocol (StemCell Technologies, Canada). The purity of the CD4+ selection was assessed by flow cytometry using a FITC-conjugated anti-CD4 antibody (anti-human CD4 antibody, clone OTK4, FITC, catalog# 60016FI, StemCell Technologies, Canada) on a BD FACSCanto II flow cytometer and then analyzed using FACSDiva software (BD Biosciences, USA) at the Analytical Biomolecular Research Facility of the University of Newcastle. All samples met a minimum purity threshold of >90 %.

### RNA isolation

Total RNA was isolated from the CD4+ T cells using the miRNeasy Mini kit (Qiagen, USA) following the manufacturer’s instructions. The quality of the RNA was assessed using the RNA 6000 Nano kit on a 2100 Bioanalyzer (Agilent Technologies, USA); a RNA integrity number greater than 8 was deemed suitable for sequencing and reverse transcription quantitative polymerase chain reaction (RT-qPCR). Purity was measured on an Epoch spectrophotometer (BioTek, USA), and concentration was measured using the high-sensitivity RNA kit on Qubit 2.0 Fluorometer (Life Technologies, Thermo Fisher Scientific, USA).

### miRNA sequencing and analysis

A cohort of 12 SPMS and 12 HC samples was run through NGS at the Diamantina Institute, University of Queensland, Brisbane, Australia. Samples were individually barcoded and then sequenced in two multiplexed pools each containing 12 samples. The sequencing libraries were prepared from 1-μg total RNA, using the TruSeq small RNA preparation kit (Illumina, USA) and sequenced using the 50-bp fragment protocol on the HiSeq 2500 platform. The sequencing generated four to nine million reads per sample, more than sufficient for expression and discovery applications. The sample sequencing reads were demultiplexed using the CASAVA 1.8 software package (Illumina, USA). The Illumina adapter sequences were trimmed from the fastq files using Trimmomatic [9]. All reads were aligned and counted against miRBase 21 [10].

### RT-qPCR

Mature miRNA TaqMan assays (Applied Biosystems, Thermo Fisher Scientific, USA) were used to determine expression of the ten most differentially expressed miRNAs in the initial NGS cohort as well as a replication cohort of 12 SPMS and 10 HC (assay IDs in miRNA numerical order: 000397, 000399, 000407, 000408, 000409, 000413, 002223, 000464, 002623, 000524). The small RNA RNU44 (ref: 001094) was used as an endogenous control. RNU44 has previously been demonstrated to be a stable control in CD4+ T cells [11], and its stability has been shown in our 47 samples (mean ± standard deviation Ct value of 23.58 ± 0.63). RNU44 was used for normalization using the ΔCt method. The relative expression (2^−ΔCt^) of all samples (24 SPMS and 22 HC) was calculated.

### Statistical analysis

The two-sample Kolmogorov-Smirnov test (K-S test) was used to test whether differences in expression levels were statistically significant between the case and control groups as implemented in R. The K-S test was chosen (over the *F* test comparison of means) because of the non-normality of the expression level distributions among miRNAs. Our statistical significance threshold allowing for multiple testing correction was determined using the False Discovery Rate (FDR) procedure of Benjamini-Hochberg [12]. Based on the number of miRNA elements, this threshold was set at 1.2 × 10^−4^. We also considered a relaxed (or nominal) significance threshold of 0.05. In addition to using statistical significance thresholds for miRNA selection, we also included a count threshold of >800 to exclude miRNAs that had very low expression levels and were unlikely to be replicated with the less-sensitive RT-qPCR. The K-S test was also used to determine significant differential miRNA and *SOCS6* expression from the RT-qPCR relative expression data.

### Correlation to patient characteristics

The Pearson correlation coefficient was calculated using RT-qPCR data for MS samples (*n* = 24) and patient characteristics: EDSS, age, disease duration, and progression duration. A correlation coefficient (*r* value) >±0.5 was considered strong, ±0.3–0.49 moderate, and <±0.29 weak.

### Gene target prediction

miRSystem integrates seven different target gene prediction algorithms and contains experimentally validated data on miRNA:mRNA interactions [13]. This integration system was used to identify genes that may be targeted by more than one of our identified dysregulated miRNAs.

### Analysis of SOCS6 expression

Five hundred nanograms of total RNA was reverse transcribed using high-capacity cDNA reverse transcription kits (Applied Biosystems, Thermo Fisher Scientific, USA) in 21 SPMS and 21 HC samples. qPCR was performed using an exon-spanning TaqMan probe for *SOCS6* (ref: Hs00377781_m1). Expression of *SOCS6* was determined as relative expression to the housekeeping genes *GAPDH* (ref: 4326317E) and *β-actin* (ref: 4326215E) using a ViiA 7 (Applied Biosystems, Thermo Fisher Scientific, USA).

## Results

We used NGS to establish miRNA expression profiles in CD4+ T cells from a cohort of 12 SPMS and 12 HC samples. RT-qPCR was then employed to validate differences in miRNA expression in the NGS cohort as well as a replication cohort of 12 SPMS and 10 HC samples (total 24 SPMS and 22 HC).

### NGS

**Fig. 1:**
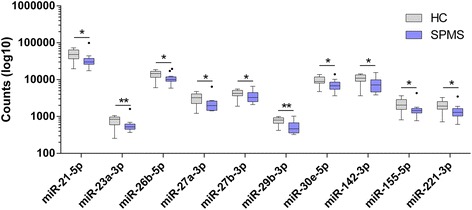
Tukey boxplot demonstrating the ten most significantly dysregulated microRNAs identified using NGS. Data is presented as log10 of the read count and clearly exhibits the down-regulation of miRNAs in SPMS (*purple*) compared to HC (*gray*). *Whiskers* represent data within 1.5 interquartile range (IQR) of the upper and lower quartile. Data points outside of the 1.5 IQR are represented by black dots. *p < 0.05, **p < 0.01

We observed three statistically significant miRNAs (miR-451a, miR-1246, and miR-144-5p) at the FDR-corrected threshold (Additional file 1: Figure S1), which probably reflects the modest sample size. These miRNAs were very lowly expressed (<100 reads per sample), and we were unable to confirm this dysregulation with RT-qPCR. We also observed 42 miRNAs at the nominal significance threshold (97 % of these were down-regulated). Of these 42 miRNAs, only 10 met our secondary criteria of having a read count >800: miR-21-5p (*p* = 0.031), miR-23a-3p (*p* = 0.007), miR-26b-5p (*p* = 0.031), miR-27a-3p (*p* = 0.031), miR-27b-3p (*p* = 0.031), miR-29b-3p (*p* = 0.007), miR-30e-5p (*p* = 0.031), miR-142-3p (*p* = 0.031), miR-155-5p (*p* = 0.031), and miR-221-3p (*p* = 0.031). Each of these miRNAs was found to be down-regulated in SPMS as summarized in Fig. 1 and was forwarded for replication testing in an independent cohort.

### RT-qPCR

**Fig. 2:**
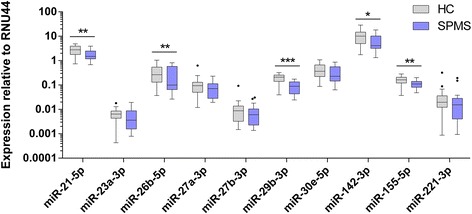
Tukey boxplot of top ten miRNAs expression (relative to RNU44) using RT-qPCR. Significant down-regulation of miR-21-5p, miR-26b-3p, miR-29b-3p, miR-142-3p, and miR-155-5p in SPMS was confirmed. *Whiskers* represent data within 1.5 interquartile range (IQR) of the upper and lower quartile. Data points outside of the 1.5 IQR are represented by black dots. *p* < 0.05, **p < 0.01, ***p < 0.001

To confirm our NGS findings, the top ten most dysregulated miRNAs were selected for further analysis in 24 SPMS and 22 HC samples using RT-qPCR (including the 12 SPMS and 12 HC samples that underwent NGS analysis). Of these ten miRNAs, RT-qPCR confirmed significant down-regulation of miR-21-5p (*p* = 0.0048), miR-26b-5p (*p* = 0.007) miR-29b-3p (*p* = 0.00001), miR-142-3p (*p* = 0.05), and miR-155-5p (*p* = 0.001) in SPMS CD4+ T cells (Fig. 2). These five miRNAs were confirmed in the original NGS cohort, the replication cohort, and the combined cohort. This provides statistically significant evidence of replication, indicating that these five miRNAs are very unlikely to be false positives. A trend of down-regulation of miRNA in SPMS samples was still observed across all ten miRNAs.

### Comparison of methods

**Fig. 3:**
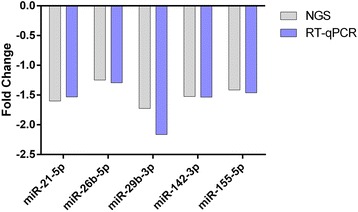
Comparison of miRNA fold-change between NGS and RT-qPCR. Magnitude of change is consistent between NGS and RT-qPCR methods

Concordance of differential expression can vary between quantitation methods [14]. To determine the magnitude of fold-change in SPMS vs. HC, we compared RT-qPCR and NGS results and found no change in the degree of decreased expression between NGS and RT-qPCR methods in the miRNAs confirmed by RT-qPCR (Fig. 3).

### Correlation to patient characteristics

No strong correlations between miRNA expression and patient characteristics were identified (Table 2). However, moderate positive correlation between EDSS and miR-21-5p, miR-26b-5p, and miR-29b-3p was seen. Further positive correlation was also found between disease duration and miR-21-5p and miR-155-5p. All miRNAs demonstrated weak correlation to patient age and progression duration.

**Table 2:** Correlation coefficients calculated from RT-qPCR data against patient characteristics

	miR-21-5p	miR-26b-5p	miR-29b-3p	miR-142-3p	miR-155-5p
EDSS	0.34	0.42	0.41	0.28	0.26
Age (HC)	0.22	0.17	0.31	0.21	−0.08
Age (SPMS)	−0.07	−0.17	−0.17	−0.30	−0.01
Disease duration	0.49	0.15	0.23	−0.08	0.49
Progression duration	0.12	0.12	0.11	−0.07	0.17

Correlation of miRNA expression and age of HC has also been calculated as a reference point for age of patients. Moderate correlations are in bold text.

### Target prediction

miRNA fold-change was <2 for all miRNAs. It is therefore unlikely that any one particular miRNA is causing a significant effect on gene expression alone. It is more likely to be a combination of multiple miRNAs targeting a few specific genes. Furthermore, as RT-qPCR is a less-sensitive methodology than NGS, and the trend of down-regulation is still observed (though not significant) in the other miRNAs, all ten miRNAs were cross-analyzed for potential gene targets. miRSystem was used to identify genes that have multiple target genes in common, both in the five confirmed miRNAs and all ten miRNAs identified by NGS. One gene, bromodomain and WD repeat domain containing 1 (*BRWD1*), is targeted by all five confirmed miRNAs. No genes are targeted by all ten miRNAs; however, eight genes are targeted by eight of the miRNAs (Table 3).

**Table 3:** Genes identified by miRSystem targeted by eight of the ten microRNAs

	miR-21-5p	miR-23a-3p	miR-26b-5p	miR-27a-3p	miR-27b-3p	miR-29b-3p	miR-30e-5p	miR-142-3p	miR-155-5p	miR-221-3p
*ACVR2B*	V	V	V	V	V	V			V	V
*ZBTB41*	V	V		V	V	V	V	V	V	
*BRWD1*	V	V	V			V	V	V	V	V
*CAMTA1*	V	V		V	V	V	V		V	V
*CFL2*	V	V		V	V	V	V	V	V	
*SOCS6*	V	V	V	V	V		V	V	V	
*MIER3*		V	V	V	V	V	V		V	V
*KLF12*	V	V	V	V	V	V	V			V

Verified targeting miRNAs are identified with a “V”

These genes are involved in transmembrane ligand binding, regulation of actin filaments, or are transcription factors. However, only one gene is specifically linked to immune cell function, *SOCS6* (suppressor of cytokine signaling 6). This gene has previously been reported to negatively regulate T cell activation by promoting ubiquitin-dependent proteolysis [15] and was consequently selected for further investigated.

### SOCS6 expression

**Fig. 4:**
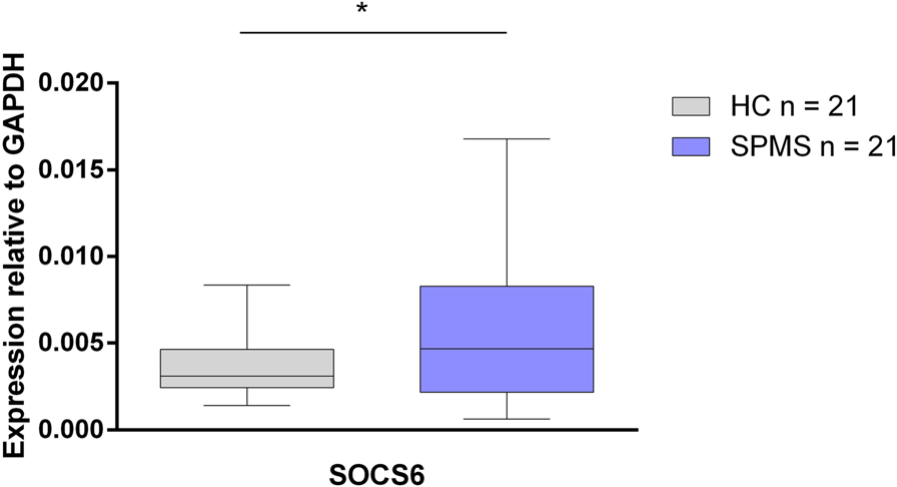
Expression of *SOCS6* relative to *GAPDH*. Up-regulation of *SOCS6* in SPMS is significant though widely distributed (*p = 0.042)

Gene expression analysis using RT-qPCR was conducted to determine whether *SOCS6* is up-regulated in SPMS CD4+ T cells in direct negative correlation to the miRNA expression (Fig. 4). Both the preliminary and validation cohorts were analyzed, and *SOCS6* expression is increased in SPMS compared to HC. Normalization against *GAPDH* and *β-actin* generated the same results (data for β-actin not shown).

## Discussion

This is the first study in MS to utilize NGS for miRNA expression profiling in the CD4+ T cells of SPMS patients. We found 42 miRNAs that are dysregulated in the CD4+ T cells of SPMS patients as compared to controls: 97 % of which were down-regulated. TaqMan assays confirmed five of these miRNAs (miR-21-5p, miR-26b-5p, miR-29b-3p, miR-142-3p, and miR-155-5p) to be down-regulated in SPMS. Each of these miRNAs (excluding miR-26b) has been reported on previously in MS though not necessarily in SPMS or CD4+ T cells. Lindberg et al. [11] identified seven miRNAs dysregulated in CD4+ T cells from RRMS patients but did not identify dysregulation in any of the five miRNA in this study. Thus, down-regulation of these miRNAs may be exclusive to SPMS.

Here, we report a decrease in miR-155-5p expression in MS. miR-155-5p has a pro-inflammatory role in MS and is up-regulated in a number of tissues. Studies of postmortem brain tissue find a gradient of miR-155-5p expression that peaks in active lesions [16] and associated neurovascular units [17] and decreases through chronic lesions and normal appearing white matter to a low baseline in healthy control (non-MS) white matter [17, 18]. This increased expression of miR-155 has been associated with the suppression of *CD47* in active lesions that creates a permissive environment for myelin phagocytosis [16]; focal adhesion and cell-cell complex molecules in the blood-brain barrier, thus increasing permeability [17] and; *AKR1C1* and *AKR1C2*, essential for biosynthesis of allopregnanolone (a neuroprotective steroid) [18].

Interestingly, a study of miR-155 in the EAE mouse model found that miR-155 expression in CD4+ T cells increases during EAE and that miR-155^−/−^ mice had an attenuation of EAE [19]. Specifically, Th17 cells lacking miR-155-5p are unable to cause EAE [20]. miR-155-5p is required for normal immune function [21], and together, these studies confirm that the significant role miR-155-5p over-expression plays in the inflammatory process of MS. In contrast, our finding of miR-155-5p down-regulation may be exclusive to SPMS patients and/or CD4+ T cells and is consistent with SPMS as a non-inflammatory mediated disease.

miR-155-5p and miR-142-3p have been identified as dysregulated in RRMS PBMCs [22], and a recent study on autologous hematopoietic stem cell transplant (AHSCT) also found co-dysregulation of miR-155-5p and miR-142-3p [23]. Contrary to our results, Arruda et al. found these miRNAs to be up-regulated in MS patient CD4+ T cells before treatment (cohort was 75 % SPMS). However, AHSCT is most effective in active MS disease, and six of the 19 SPMS patients enrolled in the Arruda et al. study presented with gadolinium-enhancing lesions in the year approaching the treatment indicating inflammatory activity. Further, the average disease duration in the Arruda et al. study was 8.1 years, as opposed to 25.6 (primary cohort) or 18.6 (replication cohort) years in our study. Our data is corroborated further by NGS expression analysis, which is a more sensitive measure of expression changes.

In a study of potential biomarkers in Alzheimer’s disease (AD), miR-26b-5p was shown to be down-regulated in the serum and CSF of AD patients when compared to patients with inflammatory neurological diseases [24], supporting the predominantly neurodegenerative pathology of SPMS. Over-expression of miR-29b insystemic lupus erythematosus (SLE) has been linked to hypomethylation of DNA in CD4+ T cells [25]. While there are currently no studies on DNA methylation in SPMS, it would be interesting to see if the down-regulation of miR-29b that we have identified here in CD4+ T cells is associated with genome-wide hypermethylation in SPMS.

Increased miR-21-5p promotes differentiation of Th17 cells in the EAE mouse model, and miR-21-5p knock-out mice are resistant to EAE [26]. Fenoglio et al. found increased miR-21-5p expression in RRMS (active relapse phase) PMBCs compared to controls, though no difference in SPMS. Again, this may be attributed to the relatively small sample size (*n* = 6) [27].

Also of interest, we previously reported miR-20a-5p down-regulation in the whole blood of all MS subtypes [28]. This miRNA was one of the 42 dysregulated miRNAs identified by NGS and is significantly down-regulated in SPMS compared to HC. However, it narrowly missed the 800 read cut-off for qPCR confirmation. miR-20a-5p is also predicted to target *SOCS6*.

Eight of the top ten dysregulated miRNAs were predicted to target *SOCS6* using MirSystem. Consistent with this, increased expression of *SOCS6* in the SPMS cohort is in direct negative correlation with the miRNA expression profiles, strongly indicating a mRNA:miRNA relationship. To our knowledge, this is the first study to identify *SOCS6* as a gene of interest in MS. It is a highly conserved gene with very low expression levels in healthy thymus and brain tissues and is down-regulated in gastric, colorectal, and pancreatic cancers [29–32]. In colorectal cancer, methylation changes have been ruled out as the mechanism of down-regulation [31]; therefore, down-regulation may be due to altered miRNA expression. MiR-424-5p is responsible for the down-regulation of *SOCS6* in pancreatic cancer [32]; however, we found no differences in miR-424-5p expression between SPMS and HC in this study.

The function of *SOCS6* as a negative regulator of T cell activation [15] and its observed over-expression in SPMS CD4+ T cells supports the notion of reduced immune activity in SPMS. Very little is known about *SOCS6*, and more studies are required to determine if it may be a novel therapeutic target.

This is the first study to use NGS miRNA profiling to assess miRNA expression in the CD4+ T cells of SPMS patients. Future studies should focus on using the same technique in treatment naïve RRMS patients to determine if this is a SPMS exclusive trend and remove the confounding factor of treatment effects. Furthermore, miRNA expression profiles of other cell subtypes should be investigated, as whole blood analysis is likely masking significant changes in individual cell subsets. Ideally, all of our patients would have had inactive SPMS; however, as SPMS is a difficult disease stage to define and collect, we have included some active SPMS patients in this study. In this study, we chose to focus on CD4+ T cells as they are thought to be the main cell infiltrates. Our previous studies also show that CD4+ T cells exhibit significant changes in methylation profiles in RRMS [33, 34].

## Conclusions

Here, we have shown a general down-regulation of miRNAs in CD4+ T cells compared to HC, with five miRNAs confirmed as significant in two independent assays. This indicates that miRNA expression may be over-normalizing in SPMS CD4+ T cells. *SOCS6* is a predicted target of the majority of these miRNAs and, consistent with this, we found *SOCS6* to be up-regulated in this cohort. These are novel findings that point towards a diminished role for CD4+ T cells in SPMS and add further evidence for SPMS being a neurodegenerative disease stage, not an inflammation-driven one.

## Additional file

Additional file 1: Figure S1. Volcano plot of differentially expressed miRNAs identified with NGS. The FDR-corrected significance threshold is demarked with a green line at *p* < 1.2 × 10^−4^. Three miRNAs were identified at the threshold. Mean read counts were low in all three miRNAs: miR-451a (SPMS mean = 76.3, HC mean = 18.9), miR-1246 (SPMS mean = 94.9, HC mean = 51.9), and miR-144-5p (SPMS mean = 15.1, HC mean = 5.5). Differential expression could not be replicated with RT-qPCR. (PNG 57 kb)

## Abbreviations

AD: Alzheimer’s disease
AHSCT: Autologous hematopoietic stem cell transplant
CNS: Central nervous system
DNA: Deoxyribonucleic acid
EAE: Experimental autoimmune encephalitis
EDSS: Expanded disability status scale
FDR: False discovery rate
GA: Glatiramer acetate
HC: Healthy controls
K-S test: Kolmogorov-Smirnov test
miRNA: MicroRNA
MS: Multiple sclerosis
NGS: Next-generation sequencing
PBMC: Peripheral blood mononuclear cells
RNA: Ribonucleic acid
RRMS: Relapsing remitting MS
RT-qPCR: Reverse transcription quantitative polymerase chain reaction
SD: Standard deviation
*SOCS6*: Suppressor of cytokine signaling 6
SPMS: Secondary progressive MS
