# Supplementary figures and images for: Next-generation sequencing reveals broad down-regulation of microRNAs in secondary progressive multiple sclerosis CD4+ T cells
Source: Clin Epigenetics. 2016 Aug 27;8(1):87. doi: 10.1186/s13148-016-0253-y (PMC5002332; doi:10.1186/s13148-016-0253-y)

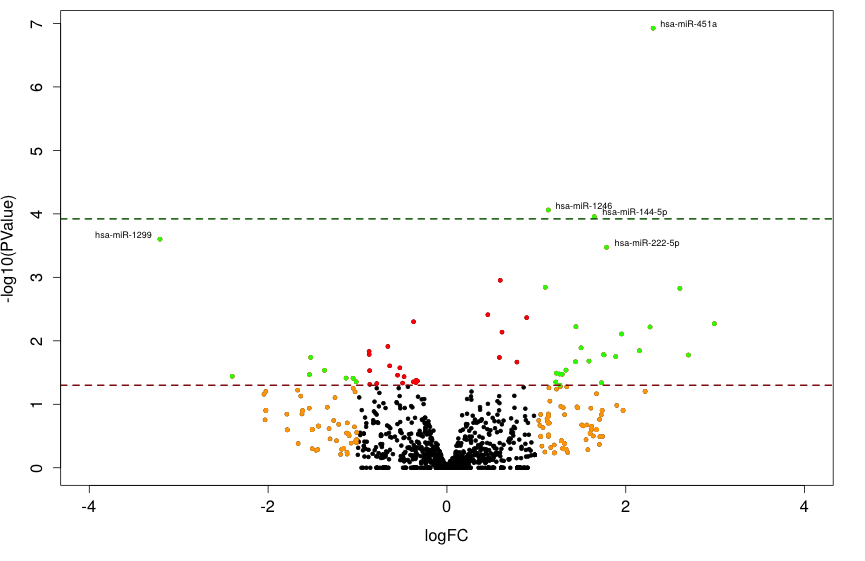

Supplement: Additional file 1: Figure S1. — Volcano plot of differentially expressed miRNAs identified with NGS. The FDR-corrected significance threshold is demarked with a green line at p < 1.2 × 10^−4^. Three miRNAs were identified at the threshold. Mean read counts were low in all three miRNAs: miR-451a (SPMS mean = 76.3, HC mean = 18.9), miR-1246 (SPMS mean = 94.9, HC mean = 51.9), and miR-144-5p (SPMS mean = 15.1, HC mean = 5.5). Differential expression could not be replicated with RT-qPCR. (PNG 57 kb) [file 13148_2016_253_MOESM1_ESM.png]
